# Influence of malocclusion on oral health-related quality of life in children: a seven-year cohort study

**DOI:** 10.1590/2177-6709.26.2.e2119244.oar

**Published:** 2021-04-30

**Authors:** Jocelito TONDOLO, Jessica Klöckner KNORST, Gabriele Rissotto MENEGAZZO, Bruno EMMANUELLI, Thiago Machado ARDENGHI

**Affiliations:** 1 Universidade Federal de Santa Maria, Departamento de Estomatologia, Faculdade de Odontologia (Santa Maria/RS, Brazil).; 2 Universidade Regional Integrada do Alto Uruguai e das Missões - URI (Erechim/RS, Brazil).

**Keywords:** Children, Cohort study, Malocclusion, Quality of life, Risk factor

## Abstract

**Objective:**

To assess the influence of early childhood malocclusion on oral health-related quality of life (OHRQoL).

**Methods:**

7-year cohort study involving 639 preschoolers (1 to 5 years) who had been evaluated initially with a survey conduced in 2010. Children completed the Brazilian version of the Child Perception Questionnaire (CPQ8-10) to assess OHRQoL during the follow-up period. Exploratory variables were collected at baseline, including the presence and severity of malocclusion (overjet and lip coverage). Socioeconomic characteristics, oral health behavior, and patterns of dental attendance were also investigated. A multilevel Poisson regression model was used to fit the association between malocclusion and OHRQoL. With this approach, incidence rate ratio (IRR) and 95% confidence intervals (95% CI) were calculated.

**Results:**

A total of 449 children were re-evaluated (follow-up rate, 70.3%). The prevalence of accentuated overjet and inadequate lip coverage was 13.5% and 11.9%, respectively. The mean (±SD) CPQ8-10 score was 10.57±10.32. The presence of inadequate lip coverage was associated with higher overall mean CPQ8-10 scores (IRR 1.51; 95% CI 1.29-1.77), and social well-being, emotional well-being, and functional limitation domains. Children with accentuated overjet (>3mm) also demonstrated higher overall scores on the CPQ8-10 than their normal counterparts. The presence of this condition also influenced the oral symptom (IRR 1.29; 95% CI 1.08-1.53) and emotional well-being (IRR 1.30; 95% CI 1.02-1.66) domains.

**Conclusion:**

Results of the present study suggest that early childhood malocclusion is a risk factor for low OHRQoL in future.

## RESUMO

**Objetivo:**

Avaliar a influência da má oclusão na primeira infância na qualidade de vida relacionada à saúde bucal (QVRSB).

**Métodos:**

Este estudo de coorte de 7 anos envolveu 639 pré-escolares (1 a 5 anos) que foram avaliados inicialmente em um levantamento transversal conduzido em 2010. As crianças completaram a versão brasileira do *Child Perception Questionnaire* (CPQ8-10) para avaliar sua QVRSB no período do acompanhamento. Variáveis exploratórias foram coletadas na linha de base, incluindo a presença e severidade de má oclusão (sobressaliência e cobertura labial). Características socioeconômicas, hábitos de saúde bucal e padrões de assistência odontológica também foram investigados. Um modelo de regressão de Poisson multinível foi utilizado para medir a associação entre má oclusão e QVRSB. Com essa abordagem, calculou-se a razão de taxa de incidência (IRR, *incidence rate ratio*) e o intervalo de confiança de 95% (IC 95%).

**Resultados:**

No total, 449 crianças foram reavaliadas (taxa de acompanhamento de 70,3%). A prevalência de sobressaliência acentuada e cobertura labial inadequada foi de 13,5% e 11,9%, respectivamente. A média±DP de pontuação do CPQ8-10 foi 10,57±10,32. A presença de selamento labial inadequado foi associada com maiores médias na pontuação total do CPQ8-10 (IRR 1,51; IC 95% 1,29-1,77) e nos domínios de bem-estar social, bem-estar emocional e limitação funcional. Crianças com sobressaliência acentuada (>3 mm) também demonstraram médias nas pontuações do CPQ8-10 total maiores do que suas contrapartes normais. A presença dessa condição também influenciou os domínios de sintomas bucais (IRR 1,29; IC 95% 1,08-1,53) e bem-estar emocional (IRR 1,30; IC 95% 1,02-1,66).

**Conclusão:**

Os resultados do presente estudo sugerem que a má oclusão na primeira infância é um fator de risco para baixa QVRSB no futuro.

## INTRODUCTION

The current definition of oral health reflects physiological, social, and psychological aspects important to the quality of life.^1^ The concept of oral health-related quality of life (OHRQoL) comprises a multidimensional and subjective perception of well-being. It is not restricted to only the physical and psychological effects of treatments, but also involves several interconnected spheres of physical, familial, and environmental questions.^2^ ^,^ ^3^ Notwithstanding, OHRQoL has been defined as a complement to clinical measurements to document the impact of oral disorders on an individual’s activities of daily life.^4^

Malocclusion is defined as a change in growth and development that affects tooth occlusion. It is considered to be a public health problem and is highly prevalent. Data from the most recent Brazilian national oral health survey revealed that approximately 37.6% of 12-year-old children exhibited some type of malocclusion.^5^ Non-aesthetic occlusal characteristics, especially in children, have been associated with unfavorable social interactions, impairing social and psychological well-being.^6^

Previous studies have assessed the association between occlusal disorders and OHRQoL of children and adolescents.^7-10^ Children with malocclusion report worse OHRQoL than their normal (i.e., non-malocclusion) counterparts.^9^ ^-^ ^11^ It occurs especially when malocclusion is located in the anterior region, such as anterior open bite and accentuated overjet.^9^ ^-^ ^11^ Despite this evidence, most studies have been cross-sectional in design, which prevents the assessment of causality. Therefore, longitudinal studies would be more useful in evaluating the influence of the cumulative effect of early malocclusion exerts on OHRQoL.^12^

Knowledge of changes in the dental transition stage could be important to the implementation of preventive approaches for children in this age group. Furthermore, it has been shown that malocclusion and its consequences are not only reflected in childhood, but may persist throughout life.^13^ Thus, the aim of the present cohort study was to assess the influence of early childhood malocclusion on OHRQoL. Our hypothesis was that children who experienced malocclusion in early childhood will report a worse OHRQoL than their normal counterparts. 

## MATERIALS AND METHODS

### STUDY DESIGN AND SAMPLE

An epidemiological oral health survey was performed in 2010 during the Children’s National Vaccination Day, in Santa Maria, Brazil. Santa Maria is a city located in southern region of Brazil. In 2010 had an estimated population of 263,403, which included 27.520 children <6 years of age. A random sample group was selected from among all children who attended health centers in the municipality on the National Children’s Vaccination Day. A total of 639 preschool children from all administrative regions of the city were orally examined by 15 examiners who were previously trained and calibrated. A multistage sampling considered all health centers with a dental office as primary survey units, and 15 out of 28 health centers were randomly selected. Each health center is responsible for vaccinating children living in that area. Caregivers of the children completed a semi-structured questionnaire designed to collect data regarding socioeconomic status, health behaviors, and the pattern of use of dental services. Details regarding the methodology followed in this first phase have been published.^14^

At follow-up (on average, 7 years later), sample planning was based on all children who were previously evaluated (n = 639). Data collection for follow-up was performed from January 2017 to March 2018 through telephone calls to schedule evaluations, and visits to children’s schools and households. After the children were located, they answered a questionnaire to assess OHRQoL. The sample size calculation accounted for an alpha error probability of 0.05, a mean score of CPQ8-10 (± standard deviation [SD]) of the exposed group (with malocclusion) of 10.9±10.7, and a mean of the unexposed group (without malocclusion) of 8.7±8.4, with a sample power of 99%.

### VARIABLES

Data regarding OHRQoL, the outcome measure of this study, were obtained at follow-up (T_2_). Previously trained interviewers applied the Brazilian version of the Child Perception Questionnaire (CPQ 8-10), which was translated and validated for Brazilian children 8 to 10 years of age.^15^ ^-^ ^17^ The CPQ8-10 comprises 25 questions divided into four domains: oral symptoms (5 questions); functional limitations (5 questions); emotional well-being (5 questions); and social well-being (10 questions). Each question has five possible answers scored on a Likert scale scored 0 to 4. Overall scores ranged from 0 to 100, with higher scores indicating worse levels of OHRQoL. 

The assessment of malocclusion, the main predictor of this study, was obtained through baseline examinations.^14^ The children were examined in health centers in dental chairs with conventional lighting, using a flat dental mirror, periodontal probe (CPI, “ball point”), and damp gauze. The variables used to measure occlusal disturbances were overjet and lip coverage. Overjet was measured in millimeters and, for analysis, was dichotomized as present (>3mm) or absent (<3mm). Lip coverage was recorded and analyzed as adequate (when the lips covered the anterior teeth completely at rest) or inadequate (when most of tooth crown was exposed and visible).^14^

Socioeconomic characteristics of the sample were recorded at baseline and included sex, annual household income, household overcrowding, mother’s education, and dental service attendance. The annual household income was collected in Real (R$-Brazilian cure - R$3.80 it was equivalent to approximately USD$1.00) and then categorized into approximate quartiles. Household crowding was evaluated according to the ratio of number of individuals to the number of rooms in a house (except the bathroom) and transformed into quartiles for analysis. Maternal schooling was collected as years of study, and categorized as completed elementary school (≥ 8 years) and those with < 8 years of education. Dental service attendance was measured according to the reason the child visited the dentist in the previous 6 months and was categorized as routine (score = 0), non-routine (score = 1), and no visit (score = 2). The feasibility of the questionnaires used was previously assessed in a sample of 20 parents during the calibration process. These parents were not part of the final sample.

### STATISTICAL ANALYSIS

Data were analyzed using STATA version 14.0 (StataCorp LLC, College Station, TX, USA). The primary outcome measure of this study were overall and domain-specific CPQ8-10 scores. The differences between participants and non-participants were assessed using the chi-squared test. Descriptive statistics were used to describe the characteristics of the sample at baseline (T_1_) and at follow-up (T_2_).

Adjusted multilevel Poisson regression models were used to fit the association between early childhood malocclusion characteristics (overjet and lip coverage) and OHRQoL at follow-up. The multilevel structure of analysis considered individuals (level 1) nested into 15 health centers (level 2). The results are presented as incidence rate ratio (IRR) and respective 95% confidence interval (CI). Variables with p-value <0.20 in the unadjusted analysis were considered in the multivariable models. 

### ETHICAL CONSIDERATIONS

The study protocol was approved by the Committee of Ethics in Research of the Federal University of Santa Maria (CAAE 54257216.1.0000.5346). All children consented to participate, and their parents or legal guardians signed an informed consent form.

## RESULTS

Of 639 children who were examined at baseline, 439 were re-examined at follow-up (70.3% follow-up rate). The reasons for non-participation were refusal to participate in the study (n=9) or the child could not be found (n=181). Comparing participants’ and non-participants’ baseline characteristics (chi-squared test), statistical differences regarding sex (*p*= 0.28), maternal education (*p*= 0.35) and overjet (*p*= 0.25) were not found. However, non-participants had a significantly higher annual income than the re-examined children (*p*< 0.05).

Demographic, socioeconomic, and oral health characteristics of the participants evaluated at baseline (T_1_) and follow-up (T_2_) are presented in Table 1. The mean age of children evaluated at baseline and follow-up was 2.8±1.4 and 10.0±1.4 years, respectively. Of the re-examined children, 229 (51%) were girls. Most of the participants in both evaluations were in the lowest household income quartiles. At baseline, the prevalence of accentuated overjet and inadequate lip coverage was 13.5% and 11.9%, respectively. 

**Table 1:** Comparison of baseline characteristics between the group of children who were followed up and the group that did not receive follow-up.

Variables	Followed-up children	Non-participants children at T_2_^a^	p*
	n (%)	n (%)	
Sex			0.28
Boys	220 (68.3)	102 (31.7)	
Girls	229 (72.2)	88 (27.8)	
Maternal education			0.35
> 8 years of formal education	246 (68.9)	111 (31.1)	
< 8 years of formal education	199 (72.4)	76 (27.6)	
Household income in R$^b^			0.03
Lowest (1^st^ quartile)	94 (68.6)	43 (31.4)	
Medium lowest (2^nd^ quartile)	129 (75.0)	43 (25.0)	
Medium highest (3^rd^ quartile)	128 (75.3)	42 (24.7)	
Highest (4^th^ quartile)	75 (61.0)	48 (39.0)	
Household crowding in people/room			0.16
Lowest (1^st^ quartile)	147 (66.5)	74 (33.5)	
Medium lowest (2^nd^ quartile)	158 (73.8)	56 (26.2)	
Medium highest (3^rd^ quartile)	34 (64.2)	19 (35.8)	
Highest (4^th^ quartile)	107 (74.8)	36 (25.2)	
Dental attendance			0.64
Routine	63 (67.0)	31 (33.0)	
Non-routine	30 (75.0)	10 (25.0)	
No visit	349 (70.4)	147 (29.6)	
Overjet			0.25
< 3mm	292 (72.1)	113 (27.9)	
> 3mm	41 (65.1)	22 (34.9)	
Lip coverage			0.47
Adequate	410 (71.0)	129 (29.0)	
Inadequate	38 (68.0)	54 (32.0)	

*p-value of chi-square test. ^a^T_2_: 7-year follow-up. ^b^R$: Brazilian Reais (R$3.80 it was equivalent to US$1.00 approximately).

Unadjusted relationships between overjet and lip coverage, with overall and domain-specific CPQ8-10 scores, are presented in Table 2. The overall CPQ8-10 scores were statistically associated with overjet (*p*< 0.05). Children who exhibited accentuated overjet and inadequate lip coverage at baseline had higher CPQ8-10 scores in the emotional well-being domain at follow-up, when compared with their normal counterparts. 

**Table 2:** Demographic, socioeconomic characteristics and oral health status of the sample.

Variables	Baseline (T_1_)^a^ (n= 639)	Follow-up (T_2_)^b^ (n= 449)
	n (%)	n (%)
Sex		
Boys	322 (50.4)	220 (49.0)
Girls	317 (49.6)	229 (51.0)
Maternal education		
> 8 years of formal education	357 (56.5)	246 (55.3)
< 8 years of formal education	275 (43.5)	199 (44.7)
Household income in R$^c^		
Lowest (1^st^ quartile)	137 (22.8)	94 (22.1)
Medium lowest (2^nd^ quartile)	172 (28.6)	129 (30.3)
Medium highest (3^rd^ quartile)	170 (28.2)	128 (30.1)
Highest (4^th^ quartile)	123 (20.4)	75 (17.6)
Household crowding in people/room		
Lowest (1^st^ quartile)	221 (35.0)	147 (33.0)
Medium lowest (2^nd^ quartile)	214 (33.9)	158 (35.4)
Medium highest (3^rd^ quartile)	53 (8.4)	34 (7.6)
Highest (4^th^ quartile)	143 (22.7)	107 (24.0)
Dental attendance		
Routine	94 (14.9)	63 (14.2)
Non-routine	40 (6.4)	30 (6.8)
No visit	496 (78.7)	349 (79.0)
Overjet		
< 3mm	405 (86.5)	292 (87.7)
> 3mm	63 (13.5)	41 (12.3)
Lip coverage		
Adequate	547 (88.1)	410 (91.5)
Inadequate	74 (11.9)	38 (8.5)

Taking into account the sampling weight. Values lower than 639 or 449 due to missing data. ^a^T_1_: baseline.

^b^T_2_: 7-year follow-up. ^c^R$: Brazilian Real (R$3.80 it was equivalent to US$1.00 approximately).

The unadjusted analysis between the malocclusion variables and OHRQoL is found in Table 3. The results of the multilevel adjusted analysis for possible confounding covariates in association with malocclusion and OHRQoL are shown in Table 4. The presence of inadequate lip coverage was associated with higher overall mean CPQ8-10 (IRR 1.51 [95% CI 1.29-1.77]), social well-being (IRR 2.05 [95% CI 1.42-2.95]), emotional well-being (IRR 1.58 [95% CI 1.13-2.20]) and functional limitation (IRR 1.94 [95% CI 1.38-2.74]) domain scores. Children with accentuated overjet (>3mm) demonstrated higher overall CPQ8-10 scores than their normal counterparts. The presence of this condition also influenced the oral symptoms (IRR 1.29 [95% CI 1.08-1.53]) and emotional well-being (IRR 1.30 [95% CI 1.02-1.66]) domains.

**Table 3:** Unadjusted association of Overall and Domain-Specific CPQ8-10 Scores at 7-year follow-up (T_2_) by the Overjet and Lip coverage. Multilevel Poisson Regression.

	n (%)	Oral symptoms	Functional limitation	Emotional well-being	Social well-being	Overall CPQ8-10
		IRRª (95% CI)^b^	IRRª (95% CI)^b^	IRRª (95% CI)^b^	IRRª (95% CI)^b^	IRRª (95% CI)^b^
Overjet						
< 3mm	405 (86.5)	1	1	1	1	1
> 3mm	63 (13.5)	1.21 (1.04-1.40)*	1.05 (0.84-1.32)	1.36 (1.11-1.67)*	0.94 (0.74-1.20)	1.16 (1.05-1.27)*
Lip coverage						
Adequate	547 (88.1)	1	1	1	1	1
Inadequate	74 (11.9)	1.14 (0.99-1.29)	0.91 (0.74-1.13)	1.24 (1.03-1.48)*	1.00 (0.81-1.24)	1.08 (0.99-1.18)

*p<0.05 ^a^IRR, incidence rate ratio. ^b^CI, confidence interval.

**Table 4:** Adjusted association of Overall and Domain-Specific CPQ8-10 Scores at 7-year follow-up (T_2_) by Overjet and Lip coverage. Multilevel Poisson Regression.

	Oral symptoms	Functional limitation	Emotional well-being	Social well-being	Overall CPQ8-10
	IRRª (95% CI)^b^	IRRª (95% CI)^b^	IRRª (95% CI)^b^	IRRª (95% CI)^b^	IRRª (95% CI)^b^
Overjet					
< 3mm	1	1	1	1	1
> 3mm	1.29 (1.08-1.53)*	0.88 (0.67-1.16)	1.30 (1.02-1.66)*	0.82 (0.61-1.01)	1.11 (0.99-1.25)
Lip coverage					
Adequate	1	1	1	1	1
Inadequate	1.11 (0.87-1.43)	1.94 (1.38-2.74)*	1.58 (1.13-2.20)*	2.05 (1.42-2.95)*	1.51 (1.29-1.77)*

*p<0.05. ^a^IRR, incidence rate ratio. ^b^CI, confidence interval. Multilevel model adjusted for sex, maternal education, household income, household crowding and dental attendance at baseline.

## DISCUSSION

The present study assessed the influence of malocclusion on OHRQoL in a cohort of children. The main finding was that malocclusion assessed according to inadequate lip coverage and accentuated overjet (>3mm) had a negative impact on children’s OHRQoL. This result supports the hypothesis that early childhood malocclusion is a risk factor for low OHRQoL over time. Our findings corroborate previous studies reporting that individuals with malocclusion experienced higher impact on OHRQoL than those without malocclusion.^8^

Individuals with accentuated overjet (>3mm) demonstrated higher overall mean CPQ8-10 scores. An analogous observation was found for the oral symptom, functional limitation, and emotional well-being domains. Other studies have reported that among the occlusal relationships evaluated, an increased overjet was considered to be the condition that most interfered with OHRQoL.^8^ Visible malocclusions, such as excessive overjet with incomplete lip coverage and diastema between the incisors, have been associated with bullying and low self-esteem among adolescents.^18^ ^,^ ^19^ However, increased overjet was not associated with social well-being domain. This result can be explained by the fact that the OHRQoL questionnaire present a multidimensional structure, which has been reported in previous studies.^15^ ^,^ ^16^ ^,^ ^20^ ^,^ ^21^ Furthermore, the OHRQoL is described as a multidimensional construct that results from an interaction between oral health conditions, social, and contextual factors.^3^ ^,^ ^15^ In this sense, despite the participation of each domain, the impact of the accentuated overjet reflects in the overall scores of the questionnaire, thus impacting a worse OHRQoL in these children.

The presence of inadequate lip coverage was associated with higher overall mean CPQ8-10 scores, and social well-being, emotional well-being, and functional limitation domain scores. It has been established that non-aesthetic occlusal aspects can induce unfavorable social responses, thus impairing social interaction and psychological well-being of the individuals affected, primarily children.^6^ Malocclusions have a large impact on OHRQoL in the social-emotional domain,^11^ while a pleasant aesthetic appearance plays an important role in social interactions and psychological well-being.^2^ Thus, it is reasonable to assume that children with untreated malocclusions can experience psychological and social consequences, thus impacting their OHRQoL. Childhood experiences have a significant impact in later years, and a negative dental appearance in childhood may be an object of provocation by other children.^22^ ^,^ ^23^

Changes in dentition occur slowly and over the developmental stages of childhood and adolescence, and are often associated with non-nutritive sucking habits, such as finger or pacifier sucking, and prolonged use of bottle-feeding, which can cause occlusal and aesthetic changes produced by unfavorable positioning of the teeth and should be avoided.^24^ To this end, the World Health Organization recommends that breastfeeding should be exclusive in the first six months, promoting improvement in the physical, mental and psychological health of children and reducing the need for non-nutritive sucking habits.^25^ It is recommended that the time limit for the elimination of pacifiers be 3 years of age.^26^ Therefore, early treatment may provide an important benefit for some children who experience teasing and negative stereotyping, and early orthodontic intervention to improve dentofacial aesthetics may improve a child’s social interactions.^24^

In addition, study psychosocial factors in early adolescence is important. Adolescence is a period of transition and it is characterized by physiological, behaviors and social changes.^27^ Through psychosocial theory, it is known that individuals with oral injuries can present physical and psychological pains and sufferings, contributing to stress, anxiety and worse OHRQoL.^28^ These consequences may interfere in health behaviors in this age group as in the practice of deleterious habits and self-care in health.^28^ Furthermore, it has been shown the changes at this stage and its consequences are not only reflected in childhood, but may persist throughout life.^13^

This study had limitations and strengths. One limitation was that we used a quantitative questionnaire. However, studies combining quantitative and qualitative measurements via questionnaires and interviews could provide more sensitive information as to how malocclusions in early childhood can affect a child’s OHRQoL years later. Future studies encompassing more malocclusion characteristics are required. In addition, for this study, data were gathering in 15 out of 28 health centers, which may affect the extern validity of the study. However, the sampling considered all health centers with a dental office as primary survey units, which were equally distributed into different areas around the city. Furthermore, the selected centers represent the major sample point in their area and accounted for nearly 85% of the children attending the vaccination program, reinforcing the representativeness of our sample. The main strengths of the present study were that it had a large cohort retention rate (70.3%) after 7 years, supporting the generalizability of our findings. Moreover, this longitudinal study assessed the influence of malocclusion on OHRQoL in a transition stage of children’s dentition, providing evidence supporting the promotion of oral health in this age group to improve of quality of life throughout life.

## CONCLUSION

Our findings support the hypothesis that anterior segment malocclusions have a significant influence on OHRQoL in children. The recognition of the changes established in the transitional stage from primary to permanent dentition, as well as the importance of preventive approaches for children in this phase of growth, when their occlusion has not yet reached maturity, makes it relevant to study this age group. An early diagnosis may facilitate the prevention of malocclusions through interceptive orthodontics, taking into account the promotion of health and OHRQoL of children.
